# Influence of Brainstem’s Area A5 on Sympathetic Outflow and Cardiorespiratory Dynamics

**DOI:** 10.3390/biology13030161

**Published:** 2024-03-02

**Authors:** Isabel Rocha, Marta González-García, Laura Carrillo-Franco, Marc Stefan Dawid-Milner, Manuel Victor López-González

**Affiliations:** 1 Lisbon School of Medicine and CCUL@Rise, Universidade de Lisboa, 1649-028 Lisbon, Portugal; 2 Department of Human Physiology, Faculty of Medicine, University of Malaga, 29590 Malaga, Spain; mgonzalezgarcia@uma.es (M.G.-G.); laura_carrillo@uma.es (L.C.-F.); msdawid@uma.es (M.S.D.-M.); manuelvictor@uma.es (M.V.L.-G.); 3 Unit of Neurophysiology of the Autonomic Nervous System (CIMES), University of Malaga, 29590 Malaga, Spain; 4 Biomedical Research Institute of Malaga (IBIMA), 29590 Malaga, Spain

**Keywords:** area A5, sympathetic nervous system, cardiovascular regulation, brainstem neurochemistry, autonomic response

## Abstract

**Simple Summary:**

Area A5 in the brain stem plays a crucial role in controlling our body’s automatic responses, especially stress, heart function and survival mechanisms. This area is re-sponsible for activating the sympathetic nervous system, which is essential for responding to stress and regulating our cardiovascular system. By understanding how the A5 area works, we can better understand how our body maintains balance and responds to changes both internally and ex-ternally. In this overview, the details of Area A5, including its structure, chemical make-up and effects on our nervous system and cardiopulmonary operations, are further explained, highlight-ing its importance to the harmony of our body systems.

**Abstract:**

Area A5 is a noradrenergic cell group in the brain stem characterised by its important role in triggering sympathetic activity, exerting a profound influence on the sympathetic outflow, which is instrumental in the modulation of cardiovascular functions, stress responses and various other physiological processes that are crucial for adaptation and survival mechanisms. Understanding the role of area A5, therefore, not only provides insights into the basic functioning of the sympathetic nervous system but also sheds light on the neuronal basis of a number of autonomic responses. In this review, we look deeper into the specifics of area A5, exploring its anatomical connections, its neurochemical properties and the mechanisms by which it influences sympathetic nervous system activity and cardiorespiratory regulation and, thus, contributes to the overall dynamics of the autonomic function in regulating body homeostasis.

## 1. Introduction

The autonomic nervous system (ANS) controls body homeostasis via a network of intrinsic neuronal plexuses and postganglionic nerves. These nerves innervate various smooth muscle structures throughout the body, including the eye muscles, the vascular system, the respiratory tract, the urinary tract, the gastrointestinal tract, the reproductive organs, the heart, the secretory and sweat glands, the hair follicles of the skin, the endocrine tissue, components of the immune system and the catecholamine-secreting cells of the adrenal medulla [1,2]. The complex and precise nature of the autonomic nervous activity is fundamental to the regulation of a variety of behavioural functions, including feeding, drinking, exercise, sexual activity, thermoregulation, micturition, defecation and important sensory processes [2,3].

The complexity of the autonomic nervous system suggests that the response to specific sensory stimuli requires customised functional and anatomical connections that originate from modality-specific sources. Current research shows that the structural organisation of the ANS is characterised by the convergence of peripheral and central afferents on organ-specific neurones at multiple levels within the central nervous system [1,2,3]. Such a configuration highlights the role of the ANS in fine-tuning physiological responses to various environmental and internal stimuli, thereby maintaining homeostasis and enabling adaptive behaviours both in health and disease conditions [4].

**Figure 1:**
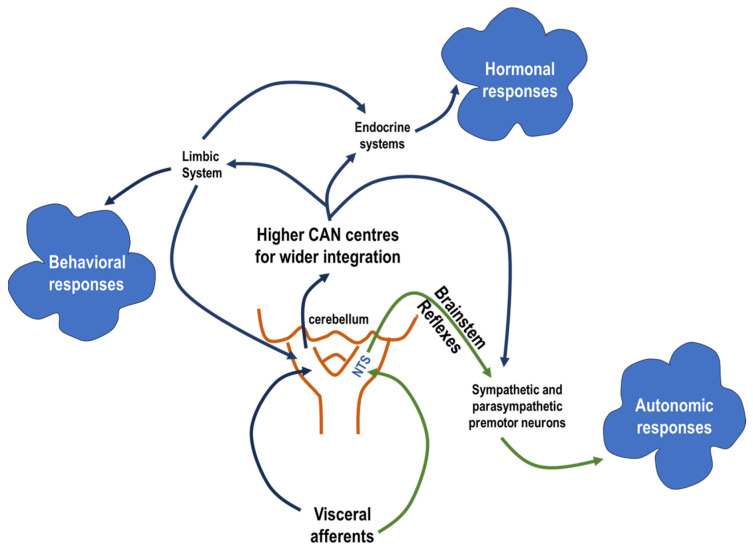
This diagram illustrates the two-level organisational structure of the autonomic reflex arc. The first level represents the simpler reflex-oriented pathways that directly modulate target organ activity in response to visceral sensory input. The second, more complex level is the central autonomic network (CAN), which integrates higher neuronal centres. This network controls a wide range of responses in the autonomic and endocrine systems as well as behavioural adaptations. The nucleus tractus solitarius (NTS) serves as a central hub where viscerosensory inputs from visceral organs are viscerotopically and functionally mapped, enabling both immediate reflexes and complex, integrated responses within the CAN.

The central component of the autonomic reflex arc has a two-level organisational structure of different complexities. The simpler level comprises reflex-orientated pathways that directly influence the target organs. The more complex level, on the other hand, involves integration with higher neuronal centres and forms a central autonomic network (CAN) that is capable of controlling comprehensive autonomic, endocrine and behavioural changes [4]. Following the reflex arc concept, viscerosensory inputs, which are transmitted by sensory fibres from visceral organs, are mapped in a viscerotopically and functionally organised manner in the nucleus tractus solitarius (NTS) (Figure 1) [1,3,4,5].

The central autonomic network itself is a complex ensemble of interconnected CNS nuclei. This network includes, among others, areas such as the parabrachial nucleus, the central grey matter, hypothalamic nuclei (lateral and paraventricular) and the central nucleus of the amygdala [1,3,6,7,8]. In a figurative analogy, CAN functions like a microprocessor, integrating various autonomic afferent signals and subsequently modulating outputs throughout the autonomic and neuroendocrine systems, potentially affecting behavioural responses in accordance with the nature of the stimuli and its influence on the homeostatic equilibrium. This complex interplay highlights the integrative ability of the CAN to maintain physiological homeostasis and respond adaptively to internal and external stimuli at any moment. The CAN concept arises from its role in processing the body’s own information and subsequently influencing specific output systems. These outputs may include hormone secretion, behaviour or changes in autonomic preganglionic neurones, with the intensity of the output response corresponding to the stimulus intensity and modality and the needed degree of homeostatic adaptation.

Sympathetic preganglionic neurones are of crucial importance as they represent the final output of the CNS within the autonomic network. These neurones play a central role in maintaining homeostasis by integrating various CNS inputs that are refined by the central processing of visceral afferent information [1,3,9]. The intricate and far-reaching nature of CAN control over sympathetic outflow is exerted via descending pathways originating in various central nervous system nuclei and highlights the complexity and precision of autonomic regulation. Brain regions identified as modulating sympathetic outflow include the paraventricular hypothalamic nucleus, noradrenergic cell group A5, caudal raphe region, rostral ventrolateral medulla (RVLM), and ventromedial medulla. Understanding the complexity of these pathways, including the neurotransmitters involved, is gaining increasing scientific attention. Expanding our knowledge of the role and function of preganglionic neurones can greatly improve pharmacological therapies and medical device development. This progress will not only improve the quality of life and well-being of patients but also pave the way for personalised medicine with a functional component that tailors treatments to individual needs and physiological responses.

## 2. Neuroanatomical Overview of Area A5

Noradrenergic neuron clusters in the brainstem act as an integrative hub, connecting different regions involved in multiple functions while receiving different afferents to coordinate responses to internal and external stimuli. The great diversity of these neurones contributes to their functional diversity. They are connected to the locus coeruleus, the brain’s main source of noradrenaline. They also project to preganglionic neurones in the spinal cord, which then synapse with postganglionic neurones. This synaptic sequence from the noradrenergic neurones in the brainstem to the preganglionic neurones in the spinal cord and then to the postganglionic neurones in the paravertebral sympathetic ganglia forms an anatomical hierarchy that is essential for physiological homeostasis.

Area A5 is, by definition, a group of catecholaminergic (noradrenergic) neurones that form a narrow column of cells located in the ventrolateral region of the parvicellular reticular formation in the caudal part of the pons [10]. It is divided into three regions based on its spatial location: lateral, dorsal and caudal. The lateral subdivision is located within the rubrospinal tract, lateral to the superior olive complex and medial to the sensory trigeminal complex. This subdivision appears to have the largest number of neurons. The dorsal subdivision is much smaller and is located in the upper part of the superior olivary complex. The caudal subdivision consists of a very small number of cells and is located lateral to the rostral pole of the facial motor nucleus. Although most neurones in area A5 are catecholaminergic neurones, there are other neurones that do not have all the typical characteristics of this neuronal type [11,12].

Area A5 was first described in the rat [13], where it appears to be well demarcated, although not so much that it has sharp boundaries with other noradrenergic groups in the pons, the A7 group and the subcoeruleus region [14]. In other species, such as cats, rabbits or dogs, area A5 is less clearly defined [15,16]. In humans, its location is very similar to that of rats, and its alterations seem to be involved in some of the cardiorespiratory manifestations observed in some pathologies, such as Rett syndrome and Ondine disease syndrome, which are currently among the aetiological factors responsible for sudden infant death syndrome [17,18,19] or being involved in the neuroinflammation and neuromodulation that mediates various neurodegenerative diseases [20,21,22,23].

The catecholaminergic neurones of area A5 possess the necessary enzymatic mechanisms that characterise noradrenergic neurones [12], which include tyrosine hydroxylase (TH), L-aromatic amino acid decarboxylase (aaDC), dopamine beta-hydroxylase (DβOH). However, a subpopulation of A5 neurones is immunoreactive for DβOH but not for the other enzymes of the cycle (TH and aaDC) [12]. These neurones are located in the most caudal levels of area A5 (approximately 600 µm rostrocaudal), whereas typical catecholaminergic neurones extend from 1.6 to 2.0 mm [13,14]. These two cellular groups also differ in their cellular shape as the typical catecholaminergic neurones of area A5 are medium-sized and often multipolar [13], and, in contrast, these non-catecholaminergic neurones are smaller, have a round soma and are often bipolar or tripolar. It has also been reported that area A5 neurones lack mRNA markers for glutamatergic, glycinergic or gabaergic transmission [24].

Pacemaker activity was also a further characteristic of the catecholaminergic neurones of area A5 [25]. The neurones of area A5 have an intrinsic activity in vitro, i.e., they are spontaneously active in pons slice preparations from newborn animals. This pacemaker activity is inhibited by presynaptic α2 autoreceptors after administration of noradrenaline [25]. The vast majority of neurones in area A5 have a low spontaneous discharge frequency between 2 and 4 discharges per second. The spontaneous activity of most cells in area A5 appears to be due to intrinsic properties rather than synaptic afferents. This auto-excitatory activity is due to irregular subthreshold oscillations in membrane potential caused by changes in voltage-activated conductance due to tetrodotoxin-sensitive sodium currents. Another feature is that spontaneous activity ceases or decreases with an increase in blood pressure. The most commonly used method of characterisation is to elicit a pressor response by intravenous administration of low doses of clonidine. They are also known to have a conduction velocity characteristic of neurones with little or no myelination (2–3 ms), i.e., they are C-fibres. All these properties are very similar to the sympathoexcitatory C1 neurones of the RVLM.

## 3. Neurofunctional Characteristics of A5 Neurones

**Figure 2:**
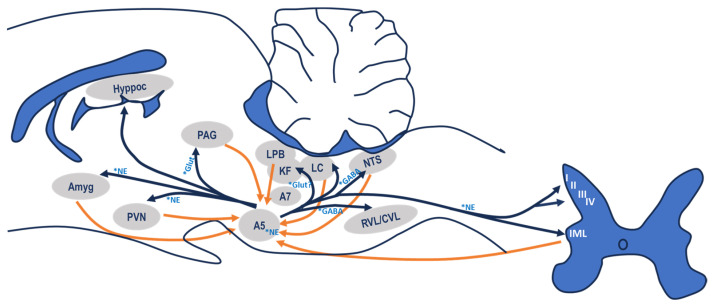
Schematic representation of the most important afferent and efferent connections of the A5 neurones within the CAN stations and the spinal cord, as well as the most important neurotransmitters involved in those connections. Amyg: amygdala; LC: locus coeruleus; LPB: lateral parabrachial nucleus, PVN: paraventricular nucleus of the hypothalamus; PAG: periaqueductal grey matter; KF: Kolliker-Fuse nucleus; NTS: nucleus of tractus solitarius; RVL/CVL: rostroventrolateral medulla/caudal ventrolateral medulla; Hyppoc: hippocampus; IML: intermediolateral cell column; NE: nor-epinephrine; Glut: Glutamate; GABA: gamma-aminobutyric acid; * refer to the main neurotransmitter of this neuronal pathway; I–IV denote the layers of spinal cord grey matter.

The connections of area A5 with other central areas have been studied in detail, although determining the physiological and/or pathophysiological consequences associated with these connections is more challenging. Area A5 has very extensive projections to many structures of the subcortical central nervous system involved in autonomic modulation (Figure 2).

### 3.1. Afferent Connections

The first study to describe the afferent connections to area A5 [26] showed that they originate from the nucleus of the solitary tract (NTS), the medullary reticular formation, the locus coeruleus, the magnocellular reticular nucleus, the parabrachial complex, the paraventricular hypothalamic nucleus and the lateral and perifornical regions of the hypothalamus [7,27,28].

Subsequently, in 1987, Byrum and Guyenet [10] published one of the most comprehensive studies to date on the afferent and efferent connections of this area. The results of this study confirmed and extended the previously described afferent connections of area A5. Area A5 receives converging afferents from the paraventricular hypothalamic nucleus, the lateral region and the dorsomedial hypothalamic nucleus, perifornical region of the hypothalamus, zona incerta, periaqueductal grey, parabrachial complex, locus coeruleus, Main sensory nucleus of the trigeminal nerve, spinal trigeminal complex, vestibular complex, intermediate and NTS caudal zones, caudal ventral area of the reticular formation, dark raphe, dorsal midline of the medulla and contralateral pontine area of the parvicellular reticular formation.

It is also clear that some areas project more densely than others to A5. Particularly dense are the projections from the intermediate and caudal zones of the NTS, the parabrachial complex, mainly from its lateral region and the Koelliker fuse and less intense from the medial region, the perifornical hypothalamic region and the paraventricular hypothalamic nucleus (especially from the posterior part of the lateral parvicellular subdivision) [28,29,30]. Interestingly, all these areas are involved in cardiorespiratory control. This morphological relationship seems to confirm the possible role of area A5 in the control and modulation of central cardiorespiratory activity. The other areas that send projections to area A5 do so in a diffuse or weak manner, so their influence is much less noticed.

Subsequent studies have completed the list of afferent connections to area A5. Of note are the connections from the periaqueductal grey matter [8,31,32], those from the neurones of lamina I of the spinal cord, from the ventral region of the medulla, more specifically from the caudal ventrolateral medulla as well as from the caudal pressor area of the caudal ventrolateral medulla [33]. Both lamina I and the caudal ventrolateral medulla region play a predominant role In the integration of nociceptive responses, adding a new role to the functionality of area A5. Afferent connections have also been described from the retrotrapezoid nucleus and the ventral respiratory group, as well as from trigeminal afferents from the nasal cavity [34].

### 3.2. Efferent Connections

At least 92% of the noradrenergic neurones in area A5 project their axons into the spinal cord, mainly to the sympathetic preganglionic neurones in the intermediolateral column of the spinal cord (cIML), in particular to lamina X and the central autonomic area [35]. This spinal projection travels through the dorsolateral funiculus to the cervical, thoracic and lumbar segments of the cIML, and its axons terminate not only in lamina X but also ipsilaterally in lamina IV, V and VI of the dorsal horn and lamina VII of the intermediate zone [35]. These data add to the known cardiorespiratory function of area A5 a possible involvement in nociceptive modulation due to the existence of reciprocal connections from area A5 to a lateral area of the caudal ventrolateral medulla and the reciprocal connections of both areas with lamina I [36,37]. The A5 group also has segmental projections to the A6 region that may influence cognition, and the synaptic connection between preganglionic A5 neurones and postganglionic noradrenergic neurones located in the paravertebral sympathetic ganglia suggests involvement in skeletal muscle regulation [38].

As for the neurones that are immunoreactive for DβH but not for TH and aaDC, their specific projections are unknown, but it is known that they do not project to the RVLM or other nuclei of the spinal cord, such as the cIML [12]. Some connections from area A5 to the ventral respiratory group have also been described [39]. In addition to the dense projection to sympathetic preganglionic neurones (92%), connections to important nuclei in mesencephalic, pontine and medullary regions have also been described [10].

Nevertheless, remarkable are the efferent bilateral connections to the medullary region, especially to the rostroventrolateral medulla, the caudal half of the NTS and the dorsal motor nucleus of the vagus [28,40,41,42]. They also show that the innervation is completely noradrenergic and that more than half of the neurones in area A5 project ipsilaterally to both nuclei, but in some cases, the projection could be bilateral. Another important efferent projection is to the region of the parabrachial complex, especially to the nucleus lateralis parabrachialis (lPB) and the nucleus koelliker-fuse (KF) [28,42]. There are also important connections with the mesencephalic area at the level of the ventral and lateral periaqueductal grey matter [8,43,44].

Following the ascending pathways, at least one-third of the neurones in area A5 project bilaterally to the hypothalamus or higher regions, with a fairly high proportion of connections extending to the tuberal level. In the hypothalamus, one-fifth of the neurones in area A5 project bilaterally to the perifornical region. This area, together with the paraventricular hypothalamic nucleus, appears to be one of the target zones for the axons of the neurones in area A5, which reinforces A5’s role in cardiorespiratory regulation. Connections with the dorsomedial hypothalamic nucleus and the paraventricular nucleus of the thalamus are also observed.

Another efferent target region for the neurones in area A5 appears to be the central nucleus of the amygdala [45], as at least one-fifth of the neurones in area A5 project bilaterally to this area, along with connections to the nucleus of the stria terminalis.

Other work has described efferent connections from area A5 to the retrotrapezoid nucleus [34], an area that has been suggested to be the location of central chemoreceptors [46]. In another work, it was described that the motor nucleus of the hypoglossus and the NTS viscerosensory nucleus receive efferent connections from area A5 in a proportion of 46% and 43% of the total catecholaminergic projections they receive, respectively, suggesting that area A5 may play an important role in the modulation of motor flow and viscerosensory transmission [41].

In summary, area A5 has particularly extensive connections at the mesencephalic, pontine and medullary levels. This entire network of afferences and efferences clearly demonstrates the potential role of this region in both cardiovascular and respiratory control.

## 4. Area A5 and Cardiovascular Regulation

Almost every region innervated by the noradrenergic neurones of area A5 plays a role in the regulation of the cardiovascular system. In addition, area A5 receives afferent signals from important cardiovascular control centres in the supraspinal central nervous system. Consequently, the group of A5 neurones is able to have a significant influence on the regulation of cardiovascular functions. This might not be surprising as the A5 group shares a common embryological origin with the Locus Coeruleus [42,43] and suggests that the two noradrenergic cell groups may represent two aspects of a single functional system, which may also include other brainstem catecholamine cell groups, each influencing unique portions of the CNS. Indeed, the A5 group may affect integrated cardiovascular response patterns by determining the global orientation of the cardiovascular regulatory system.

Nevertheless, the function of A5 neurones in the regulation of blood pressure is still a controversial topic, as different results are obtained when these neurones are activated. In fact, various authors have referred to two completely antagonistic roles in the cardiovascular system: an excitatory or an inhibitory role. In some cases, dichotomous relationships have emerged, as chemical stimulation has led to a reduction in blood pressure and bradycardia, suggesting sympathoinhibition [45], while electrical stimulation has shown sympathoexcitatory effects, although there are cases where it has even been cardioinhibitory.

Electrophysiological studies have shown that the neurons in area A5 exhibit spontaneous activity that is attenuated by elevated blood pressure, while a decrease in blood pressure increases their activity, as shown in studies [14,44,46,47]. This phenomenon suggests that the A5 neurones underlie inhibitory synapses that are crucial for the modulation of blood pressure. Huangfu and co-workers also found a direct correlation between the firing rates of A5 neurons and the electrical discharges of the splanchnic sympathetic nerve and suggested a similarity between these neurons and the excitatory neurons in the RVL that connect to the sympathetic preganglionic neurons in the CML [44]. This relationship emphasises a complex network of inhibitory control, which is complemented by the findings that RVL neurons receive consistent inhibitory input from CVL neurons [48]. In addition, Kwiat and co-workers found GABAergic synapses on noradrenergic neurons in area A5, suggesting persistent inhibition of these neurons [49].

These observations are complemented by studies showing that fluctuations in blood pressure induced by intravenous administration of vasoactive substances such as vasopressin, angiotensin II [47], phenylephrine [44], noradrenaline or sodium nitroprusside [50] correlate with changes in the intrinsic activity of A5 neurones. In particular, an increase in blood pressure leads to a decrease in A5 neuron activity and vice versa. This is confirmed by the increased expression of Fos protein in the A5 area after hypotension induced by continuous SNP infusion [51,52] or after haemorrhage [53].

In studies with non-anaesthetised animals, Ramos observed that microinjection of L-glutamate into the A5 area of awake rats resulted in a dose-dependent increase in blood pressure and tachycardia [54]. These results indicate that the A5 area, when stimulated under more physiologically appropriate experimental conditions that avoid potential interference from the effects of anaesthetics on CNS processing, functions primarily as a pressor area, suggesting an important role for this group of neurons in the control and/or modulation of sympathetic efferent activity.

In addition, Guyenet has shown that the response of A5 neurones to blood pressure changes depends on intact baroreceptive afferents, as observed in animals with severed aortic sinus nerves [47]. Given the role of baroreceptors in reducing efferent sympathetic output and lowering blood pressure, it is concluded that baroreceptor-mediated inhibition of A5 neuron activity is essential for the sympatho-inhibitory response of the baroreflex. Regarding the relationship between A5 and the baroreceptor reflex, studies in the scientific literature indicate that area A5 is also involved in the integration process of the baroreflex mechanism. Research by Coote and MacLeod in 1974 showed that the noradrenergic efferent pathways leading to CML must remain intact for the sympatho-inhibitory component of the baroreflex to be effectively expressed [55]. Given that noradrenergic cell groups in the pons have been identified as the sole suppliers of noradrenergic terminals to the spinal cord, as Westlund stated in 1983, it logically follows that area A5 is involved in the reflex responses elicited by baroreceptor stimulation [56]. In addition, Guyenet found that the neuronal firing changes in area A5 did not manifest in animals subjected to baroreceptor denervation [47]. Also, the selective destruction of the A5 noradrenergic cells with 6-hydroxydopamine (6-OHDA) alters baroreflex responses [10]. These results indicate that the neurons of area A5 are an essential component of the neuronal circuitry of the baroreflex.

In unitary neuronal recordings of A5 cells [8,25,28,57], it was observed that the activity of neurones in area A5 is strongly influenced by afferent inputs of cardiorespiratory control, such as arterial baroreceptors and peripheral chemoreceptors [57]. In addition, 63% of the recorded neurones are inhibited by an increase in arterial pressure associated with the injection of adrenaline or a baroreceptor stimulus due to aortic constriction. Regardless of the baroreceptor activation, the inhibition occurs in a range of arterial pressure extending from 80 to 140 mmHg. Similarly, during the drop in blood pressure caused by stimulation of the von Bezold-Jarisch reflex, in which activation of the ventricular receptors occurs by intravenous administration of phenylbiguanide (serotonergic agonist), there is a simultaneous decrease in sympathetic nerve discharge, which is counteracted by a strong increase of the noradrenergic neurones of area A5 which confirms their sympathoexcitatory role.

Over the last 20 years, in several reviews of sympathetic control of the cardiovascular system and the integration mechanisms of baroreceptor and chemoreceptor reflexes and the central pathways that support them, both in conscious animals and under anaesthesia, several authors agree in assigning an important role to area A5 in sympathoexcitatory control [58,59,60,61,62]. In addition, recent work by our research group [8,28,32,62,63] highlights this sympathoexcitatory activity by demonstrating the involvement of area A5 in the defence response elicited by the hypothalamic defence area and the dorsolateral periaqueductal grey matter. In all these studies, we were able to demonstrate, for the first time and using electrophysiological, neuroanatomical and neuropharmacological techniques, the existence of functional connections between these regions. It appears that area A5 is functionally essential for the autonomic changes that occur during the activation of the defence response from these regions, including the inhibition of the baroreceptor reflex. Without this inhibition, the increase in heart rate and blood pressure necessary for the effective development of the animal’s defence response could not be achieved. It is assumed that the involvement of area A5 in the defence response is mediated by afferent connections from both the hypothalamic defence area and the dorsolateral PAG. This is supported by the connections that both regions have with the parabrachial complex, as well as area A5’s own efferents to the medulla (NTS and RVLM) and spinal cord (cIML) areas involved in this response.

Recently, optogenetic targeting of A5 neurones in anaesthetised rats has shown that their activation does not alter blood pressure but causes a brief increase in renal and lumbar sympathetic nerve activity, which suggests that A5 neurones primarily stimulate visceral sympathetic nerve activity, having less influence on skeletal muscle sympathetic activity and on blood pressure [30,63]. But the role of noradrenaline makes things even more complex as it can both excite and inhibit sympathetic preganglionic neurones, this effect being dose-dependent. In addition, the used animal species might also interfere as the sympathetic excitation and or inhibition might have different expressions in accordance with it. For instance, the paradigmatic defence reaction is perceived with an alert reaction in the rat and with a playing dead reaction in the rabbit, the most used species in laboratory studies for A5 function. Nevertheless, later, in an in-vivo freely moving study in rats, stimulation of A5 neurones was primarily shown to increase splanchnic sympathetic nerve activity and lead to a significant increase in systemic blood pressure [63].

In conclusion, recent research indicates a predominantly sympathoexcitatory role of area A5 neurons in the regulation of cardiovascular function. Despite initial controversy and the observation of both sympathoexcitatory and sympathoinhibitory effects, current evidence, including consistent neuronal recordings and optogenetic studies, suggests that area A5 plays an essential role in the modulation of blood pressure and heart rate. This role is particularly pronounced in the context of chemoreceptor reflexes. However, the complexity arising from the different effects of noradrenaline and the variations in different animal models emphasises the need for further studies to fully clarify the functions of area A5 in cardiovascular regulation.

## 5. The Role of Area A5 in the Control of Breathing

Breathing is controlled by a network of neurones in the brainstem that are responsible for generating rhythms, modulating movement patterns and monitoring physiological states [64].

It was demonstrated some years ago that noradrenergic groups in the brainstem are involved in breathing control as follows: (1) endogenous noradrenaline released by the four noradrenergic groups in the brainstem promotes (A1/C1 and A6) or reduces (area A5) the activity of the central respiratory rhythm generator. (2) areas A5 and A6 exert a double antagonistic effect at the medullary level, with area A5 having an inhibitory effect on the respiratory rhythm by activating α2 receptors, while area A6 accelerates the rhythm by activating α1 receptors. (3) pontine area A5 and the medullary A1/C1 group exert their antagonistic effect on the central respiratory rhythm generator through the activation of α2 receptors, although it is not known whether there are two functionally distinct types of α2 receptors. (4) the medullary A2/C2 group may play a normalising role on the central respiratory rhythm generator through mechanisms and receptors that are not yet fully defined. All these findings suggest that the neurones in area A5 establish a bidirectional modulatory control with the central respiratory neurones and, therefore, play an important role in respiratory control in different physiological situations [44,65,66,67].

There are only a few studies that have attempted to clarify the impact of area A5 on respiratory control. Whenever the ventrolateral pontine area has been investigated, it has been conducted only superficially and usually as an adjunct to the investigation of cardiovascular activity. In fact, the possible role of the pons in respiratory control was investigated using techniques to transect the bulbopontine nerve [66]. In this study, which has become a classic, the author demonstrated that certain pontine structures, known as pneumotaxic centres, are involved in the maintenance of normal breathing patterns in anaesthetised, vagotomised adult cats. Furthermore, transection of the middle pontine area, removing the efferents of the rostral pons, resulted in a specific pattern called “apnoeic”, characterised by long, sustained inhalations interspersed with short exhalations. This pattern of breathing required not only the absence of certain pontine structures but also vagotomy and anaesthesia. Electrical stimulation, electrolytic lesions and neuronal recordings have classically localised the pneumotaxic centres to the medial parabrachial nucleus and the Kolliker-Fuse nucleus, involved in the fine-tuning of the respiratory rhythm and contribute to the transition between the phases of the respiratory cycle and the pontine respiratory group which interacts with the medullary respiratory centres (such as the pre-Boetzinger complex, which is crucial for the generation of the respiratory rhythm) to modulate the depth and frequency of breathing [67]. Furthermore, it is known that the respiratory control neurones of these nuclei exert their function via glutamate acting on NMDA receptors of the target cells, as apnoea can be produced by blocking these receptors [68].

Later, the possibility that central noradrenergic neurones could produce this type of breathing pattern originating from the pneumotaxic centre by producing apnoea through inhibition of the locus coeruleus was introduced. Over the years, it has been speculated that these centres may not exist in rats, as the doses of NMDA antagonists used were not comparable to those effective in cats [69]. Recently, the possibility that other nerve centres are involved has been considered. Chemical lesions in regions of the ventrolateral pons, including area A5, result in an apnoeic breathing pattern in anaesthetised, vagotomised, paralysed and artificially ventilated adult rats [17,28,70]. This knowledge leads to a deeper investigation of the role of area A5 in respiratory regulation. Early work confirmed that broad stimulation of the ventrolateral pons, including area A5, with glutamate prolongs expiration [11]. Various degrees of apnoea have also been described following electrical stimulation of area A5 [71]. In addition, the possible role of this area has been investigated with “in vitro” studies on pontine-medullary slices of neonatal specimens [72]. In these in vitro neonatal pontine-medullary preparations of rats, local electrolytic lesions of area A5 activated the medullar respiratory rhythm generator and increased the frequency of phrenic discharge. On the other hand, electrical stimulation of area A5 inhibited it by decreasing the respiratory rate due to an increase in expiratory time [72].

Our group performed the final confirmation of the role of area A5 in cardiorespiratory control. The results of the study indicate that the respiratory response to specific activation of the somas of area A5 by microinjection of glutamate consists mainly of a decrease in respiratory rate due to a shortening of the expiratory time [70]. These data suggest that the respiratory response induced by microinjection of glutamate is due to activation of the somas in area A5. However, it is known that any increase in arterial pressure reflexively causes a concomitant decrease in respiratory rate. To clarify the actual role of area A5 neurones, electrical and glutamate stimulation of area A5 neurones was performed after blocking the pressure response by administration of guanethidine, leading to a decrease in respiratory rate and phrenic activity in anaesthetised, paralysed and artificially ventilated animals. This appears to be the actual response pattern of area A5 neurones when they are directly activated and when the main cardiorespiratory reflexes are excluded or blocked.

Subsequent studies in mice repeat the techniques used previously and add the application of noradrenergic agonists to the respiratory rhythm generator, also achieving a decrease in respiratory rate [73]. That is, they confirm that the electrolytic lesion of area A5 activates the respiratory rhythm generator, while the application of noradrenergic agonists inhibits it, showing that area A5 neurones are responsible for the noradrenergic inhibition received by the medullary respiratory rhythm generator via α2 receptors although this inhibition is not strong enough in rats to abolish respiratory rhythmogenesis, which does occur in mice [68].

The possible role of area A5 in respiratory control is also suggested by morphological studies in adult rats using microinjection of trans-synaptic viruses into phrenic motor neurones. These studies have shown that area A5 contains third-order neurones involved in the neuronal network that controls diaphragmatic activity and, thus, phonation and breathing [71]. In addition, other studies on newborn rats add some light to A5 on respiratory control in the neonate whose respiratory control system is initially underdeveloped and undergoes extensive maturation in the postnatal phase, which is characterised by a decrease of the number of neurones in the A5 region until the 10th day [74] followed by stabilisation of the number of A5 noradrenergic neurones from the third month onwards [60]. Results showed that neonatal noradrenergic A5 neurones are not involved in the maintenance of baseline ventilation or in mediating ventilatory responses to increased carbon dioxide conditions. However, these neurones play a critical role in regulating respiratory drive under hypoxic conditions, which is essential for maintaining stable respiratory function in low-oxygen environments in early life [60].

Furthermore, the neurones of area A5 have a respiratory discharge profile and mostly have post-inspiratory activity, which is also involved in the respiratory responses to hypoxia and hypercapnia [61,75,76]. When the hypoxic stimulus is withdrawn, the respiratory rate falls below the initial level. This phenomenon, associated with an increase in expiratory time, is referred to as a “posthypoxic decrease in respiratory rate” and was abolished for the first time through electrolytic lesions of area A5 and after microinjection of muscimol (GABAergic agonist) into the same region [75]. Later, these authors confirmed that the decrease in respiratory rate is caused by activation of the expiratory neurones of the brainstem and that glutamatergic neurotransmission at the level of area A5 is involved in the modulation of the “posthypoxic decrease in respiratory rate [77]. In another study, these results were confirmed through the lesion of area A5 and exposure of the animals to hypoxia and hypercapnia, which led to the abolition of the “posthypoxic respiratory frequency decrease” and the reduction of the respiratory response to hypercapnia after the silencing of area A5 neurones [76].

In summary, the research emphasises the crucial role of area A5 in the control of breathing, particularly through noradrenergic modulation of respiratory rhythms. Activation of neurons of area A5, as shown in various studies, often leads to a reduction in respiratory rate due to changes in expiratory times. In addition, area A5 is crucial for diaphragmatic control and responses to hypoxia and hypercapnia. This emphasises its essential role in both normal and pathophysiological respiratory functions within the brainstem respiratory network.

## 6. Conclusions and Future Directions

In the ventrolateral pons of the brain stem, the group of noradrenergic neurones A5 plays a decisive role in the regulation of the sympathetic and respiratory systems. These neurones, which are characterised by their slow, regular resting activity, project spinal axons into the intermediolateral cell column. The neurophysiological responses of A5 neurones include activation by nociceptive stimuli, inhibition by α2-adrenergic agonists, variable inhibition by an increase in arterial pressure, activation by chemoreceptors and some degree of respiratory modulation. The A5 region is rich in catecholaminergic axonal terminals and receives significant input from major catecholaminergic regions, and it projects to other cathecolaminergic brainstem areas as well as to the spinal cord. This connectivity suggests a potential modulatory influence of various neuronal cells at different levels of the neuroaxis and thus allows speculation of the recruitment and integration mechanisms of brainstem catecholaminergic neurones in different physiological and pathophysiological in varied contexts such as hypertension, chronic pain, changes in cognition or locomotion.

In the context of pain, bidirectional modulation of spinal nociceptive processing by noradrenergic pathways is dependent on the specific type of pain. This modulation is not only influenced by the type of nociceptive stimulus but also by other structures of the central nervous system involved in emotional, motivational, or attention-related states. As mentioned above, noradrenergic neurons in the A5 region have been shown to be a potential key centre for modulating actions of higher brain structures. These actions inhibit nociceptive transmission at the level of the dorsal horn of the spinal cord by activating presynaptic alpha2 receptors.

This overview lays the foundation for future research, but many questions remain unanswered. For example, how does the A5 region respond to functional or structural changes triggered by genetic or epigenetic factors? Are this region and its connections equally affected by different pathological conditions? Do the noradrenergic cells of this nucleus show similar responses to different external stressors, or do their functional responses differ depending on their location within the nucleus or their projections? Is the selective degeneration of A5 neurons in diseases such as multiple system atrophy and Parkinson’s disease related to the progression of dysautonomia or other dysfunctional disorders? To answer these questions and deepen our understanding of the physiological and pathophysiological role of noradrenergic pontine cell groups, more comprehensive studies that include both basic and clinical research are needed.

## Data Availability

Not applicable.
